# Diagnostic Accuracy of Early Secretory Antigenic Target-6–Free Interferon-gamma Release Assay Compared to QuantiFERON-TB Gold In-tube

**DOI:** 10.1093/cid/ciz034

**Published:** 2019-01-21

**Authors:** Elisa Nemes, Deborah Abrahams, Thomas J Scriba, Frances Ratangee, Alana Keyser, Lebohang Makhethe, Mzwandile Erasmus, Simbarashe Mabwe, Nicole Bilek, Virginie Rozot, Hennie Geldenhuys, Mark Hatherill, Maria D Lempicki, Line Lindebo Holm, Leah Bogardus, Ann M Ginsberg, Thomas Blauenfeldt, Bronwyn Smith, Ruth D Ellis, Andre G Loxton, Gerhard Walzl, Peter Andersen, Morten Ruhwald

**Affiliations:** 1 South African Tuberculosis Vaccine Initiative, Institute of Infectious Disease and Molecular Medicine & Division of Immunology, Department of Pathology, University of Cape Town; 2 AERAS, Rockville, Maryland; 3 Statens Serum Institute, Copenhagen, Denmark; 4 South Africa Department of Science and Technology–National Research Foundation Centre of Excellence for Biomedical Tuberculosis Research, South African Medical Research Council Centre for Tuberculosis Research, Division of Molecular Biology and Human Genetics, Faculty of Medicine and Health Sciences, Stellenbosch University, Cape Town

**Keywords:** tuberculosis infection, diagnosis, IFN-γ release assay, qualification

## Abstract

**Background:**

Early secretory antigenic target-6 (ESAT-6) is an immunodominant Mycobacterium tuberculosis (M.tb) antigen included in novel vaccines against tuberculosis (TB) and in interferon-gamma (IFN-γ) release assays (IGRAs). Therefore, the availability of an ESAT-6–free IGRA is essential to determine M.tb infection status following vaccination with ESAT-6–containing vaccines. We aimed to qualify a recently developed ESAT-6–free IGRA and to assess its diagnostic performance in comparison to QuantiFERON-TB Gold In-tube (QFT).

**Methods:**

Participants with different levels of M.tb exposure and TB disease were enrolled to determine the ESAT-6–free IGRA cutoff, test assay performance in independent cohorts compared to standard QFT, and perform a technical qualification of antigen-coated blood collection tubes.

**Results:**

ESAT-6–free IGRA antigen recognition was evaluated in QFT-positive and QFT-negative South African adolescents. The ESAT-6–free IGRA cutoff was established at 0.61 IU/mL, based on receiver operating characteristic analysis in M.tb-unexposed controls and microbiologically confirmed pulmonary TB patients. In an independent cohort of healthy adolescents, levels of IFN-γ released in QFT and ESAT-6–free IGRA were highly correlated (P < .0001, r = 0.83) and yielded comparable positivity rates, 41.5% and 43.5%, respectively, with 91% concordance between the tests (kappa = 0.82; 95% confidence interval, 0.74–0.90; McNemar test P = .48). ESAT-6–free IGRA blood collection tubes had acceptable lot-to-lot variability, precision, and stability.

**Conclusions:**

The novel ESAT-6–free IGRA had diagnostic accuracy comparable to QFT and is suitable for use in clinical trials to assess efficacy of candidate TB vaccines to prevent established M.tb infection.

## Teaser

The novel early secretory antigenic target-6 (ESAT-6)–free interferon-gamma release assay has comparable diagnostic performance for *Mycobacterium tuberculosis* (M.tb) infection as the QuantiFERON-TB Gold assay and is suitable for diagnosing M.tb infection in clinical trials of ESAT-6–containing tuberculosis vaccines.

Early secretory antigenic target-6 (ESAT-6) is an immunodominant antigen expressed by *Mycobacterium tuberculosis* (M.tb) [1]. ESAT-6 and its heterodimeric complex protein CFP-10 are the principal components of immunodiagnostic tests for M.tb infection, including the interferon-gamma (IFN-γ) release assay (IGRA) and novel specific skin tests [2, 3]. These small, highly immunodominant antigens are encoded in the region of difference 1 of the M.tb genome, which comprises many important virulence factors as components and substrates of the ESAT-6 system 1 (ESX1) type VII secretion system [4] and are absent from bacille Calmette-Guerin (BCG), thereby, importantly, enabling distinction of M.tb infection from prior BCG vaccination [5].

ESAT-6 is also increasingly recognized as an important vaccine antigen with unique properties for infection containment and, potentially, for clearance [6–8]. As such, ESAT-6 is contained in many candidate vaccines against tuberculosis (TB), including the subunit vaccine H56:IC31, which is undergoing phase 2 clinical testing [6, 9]. Vaccine efficacy to prevent established M.tb infection, as a likely predictor of prevention of TB disease, has been proposed as an innovative and cost-effective endpoint to rationalize TB vaccine development [10]. We recently showed that BCG revaccination or vaccination with a novel subunit vaccine candidate (H4:IC31) may offer partial protection against sustained M.tb infection, measured by QuantiFERON-TB Gold In-tube (QFT) [11]. That trial provided the first proof-of-concept efficacy signal for a subunit vaccine candidate, which is very similar to H56:IC31 but does not contain ESAT-6. Such a clinical trial design, one that relies on IGRA to measure M.tb infection, cannot currently be used to evaluate ESAT-6–containing vaccine candidates, since vaccine-induced immune responses are cross-reactive with those measured by IGRA, rendering the test false positive.

Until recently, inclusion of ESAT-6 and CFP-10 has been considered essential for adequate immunodiagnostic sensitivity. However, recent advances in antigen discovery tools and M.tb antigen recognition studies in humans point to novel diagnostic antigens that could replace ESAT-6 [12–14]. Several studies explored the diagnostic potential of new candidates either alone or in combination, and we recently established an ESAT-6–free IGRA antigen cocktail after screening of several candidates (TB7.7, EspJ, EspC, EspF, EccD1, PE35, and Rv2348) compared to ESAT-6 and CFP-10 [15].

In this study, our aim was to confirm the recognition of an ESAT-6–free IGRA antigen cocktail in adolescents from a high TB burden area, define a diagnostic algorithm for the ESAT-6–free IGRA, evaluate ESAT-6–free IGRA diagnostic performance compared to QFT, and assess the variability of ESAT-6–free IGRA blood collection tubes.

## METHODS

### Study Participants

All studies that involved human participants were conducted according to the principles described in the Declaration of Helsinki and were approved by relevant institutional review boards (Human Research Ethics Committee [HREC]). Written informed assent and consent were obtained from participants and their legal guardians (for minors).

We included the following 4 independent cohorts of patients to address the study aims (Supplementary Figure 1A). Cohort 1: Healthy adolescents (cohort 1a) and adults (cohort 1b) were enrolled at the South African Tuberculosis Vaccine Initiative (SATVI, South Africa, HREC reference: 126/2006) to confirm ESAT-6–free IGRA antigen recognition and perform ESAT-6–free IGRA qualification, respectively. Individuals who had received a tuberculin skin test (TST) in the last 12 months, had evidence of any acute or chronic disease or symptomatic infection (including human immunodeficiency virus [HIV] positive or TB symptoms), currently used immune-modifying drugs, had been enrolled in any experimental study, were pregnant or lactating (women), weighed <50 kg, or those with raised suspicion of anemia by recruiting officer were excluded. Cohort 2a: Healthy adults with no known exposure to M.tb were recruited at the Statens Serum Institut (SSI) in Denmark (HREC reference: H-3-2012-008). Individuals with a medical history of M.tb exposure, travel, and/or risk-seeking behavior were excluded. Cohort 2b: Adult patients with pulmonary TB disease were recruited by the Immunology Research Group (IRG) at Stellenbosch University, South Africa (HREC reference: N13/05/064). TB disease was microbiologically confirmed by sputum liquid culture and/or GeneXpert and/or smear microscopy. Patients with HIV infection, multidrug-resistant TB, or those who started TB treatment more than 3 days before enrollment were excluded. Cohort 3: A subset of healthy adolescents screened for enrollment in a prevention-of-M.tb-infection trial at SATVI (HREC reference: 471/2013; clinicaltrials.gov identifier: NCT02075203) [11] were included in this study to evaluate ESAT-6–free IGRA performance compared to QFT.

Inclusion and exclusion criteria for cohort 3 have been described in detail elsewhere [16]. QFT results from cohort 2a, 2b, and 3 have been published in a different form [16].

### Assessment of Preselected Antigens

Venous blood was collected in lithium heparin–containing vacutainers, and 1 mL per tube was dispensed in stimulation tubes (Sarstedt, Numbrecht, Germany) that contained (a) no antigen, or pools of overlapping 15–20 mer peptides representing: (b) full length CFP-10, (c) a fragment of EspC (aa54-103), (d) a fragment of EspF (aa9-44), (e) a fragment of Rv2348 (aa56-108), or (f) ESAT-6–free IGRA antigen cocktail (b+c+d+e, at a concentration of 5 μg per peptide/mL blood, as described [15]). Phytohemagglutinin (Dartford, Remel, United Kingdom) was used as the positive control (5 μg/mL), and a set of QFT tubes (Nil, TB antigen, mitogen; Qiagen, Hilden, Germany) was run in parallel as the reference. Within 2 hours of phlebotomy, stimulated blood was incubated at 37°C for 18–22 hours and then centrifuged. Plasma was harvested and cryopreserved at –80°C until batch analysis.

### QFT and ESAT-6–Free IGRA Assays

Blood was collected directly in QFT tubes and in tubes that contained lyophilized ESAT-6–free IGRA peptides, manufactured at SSI, as described [15], to define a cutoff for the ESAT-6–free IGRA assay, determine assay performance in adolescents, and assess tube variability. Blood was processed following a standard operating procedure that was stricter than that recommended by the manufacturer in order to reduce known sources of assay variability and improve reproducibility, as described [16]. Briefly, 1 mL of blood was collected and mixed by 10 manual inversions and 5 minutes on a tube rotator and incubated at 37°C within 2 hours of phlebotomy, for 16–20 hours.

To determine the ESAT-6–free IGRA assay cutoff, blood was collected and processed at SSI (M.tb unexposed controls, cohort 2a) and IRG (TB patients, cohort 2b). Cryopreserved plasma was analyzed by QFT IFN-γ enzyme-linked immunosorbent assay (ELISA) at SATVI.

To assess the performance of ESAT-6–free IGRA in South African adolescents, samples were collected and processed at SATVI. Blood was simultaneously collected and processed for the QFT and ESAT-6–free IGRA. As results from QFT analysis were part of the screening procedures for real-time enrollment in a clinical trial [11], fresh plasmas harvested from QFT tubes were analyzed by QFT IFN-γ ELISA, while plasmas harvested from ESAT-6–free IGRA tubes were cryopreserved and analyzed in batches. We have previously shown that QFT results obtained from fresh vs frozen plasma are comparable [16].

### ESAT-6–Free IGRA Tube Technical Qualification

Blood was collected from participants enrolled in cohort 1b directly in QFT and ESAT-6–free IGRA tubes (Supplementary Figure 1B) and processed, as described [16]. Identical internal quality control samples were included in each plate to assess interassay variability.

### IFN-γ ELISA

IFNγ was quantified by QFT IFN-γ ELISA (Qiagen, Hilden, Germany). All samples from the same participant were included in the same plate, and each sample was analyzed in triplicate or duplicate wells by a blinded operator. Results from replicate wells were averaged unless a technical error that affected 1 of the wells was recorded.

### Statistical Analyses

Statistical analyses were performed using SAS 9.2 (SAS Institute) and GraphPad Prism (V7, GraphPad Software Inc.). IFN-γ values were compared using nonparametric methods across study groups (Mann-Whitney test) or assays (Wilcoxon matched-pairs signed rank test or Spearman correlation). Diagnostic accuracy and assay cutoff were determined by receiver operating characteristic analysis. Assay sensitivity and specificity for a given cutoff are reported with Clopper-Pearson 95% confidence intervals (CIs). Agreement and the marginal homogeneity between 2 assays were calculated using Cohen’s kappa and the McNemar test, respectively. Assay variability and bias were calculated using Bland-Altman analysis.

## RESULTS

### Recognition of ESAT-6–Free IGRA M.tb Antigens by South African Adolescents

Since M.tb antigens included in the ESAT-6–free IGRA were originally selected in participants from low and intermediate TB burden settings [15], we first confirmed their recognition in adolescents from a high TB burden area who represent a target population for prevention of M.tb infection trials (cohort 1a).

**Figure 1.**
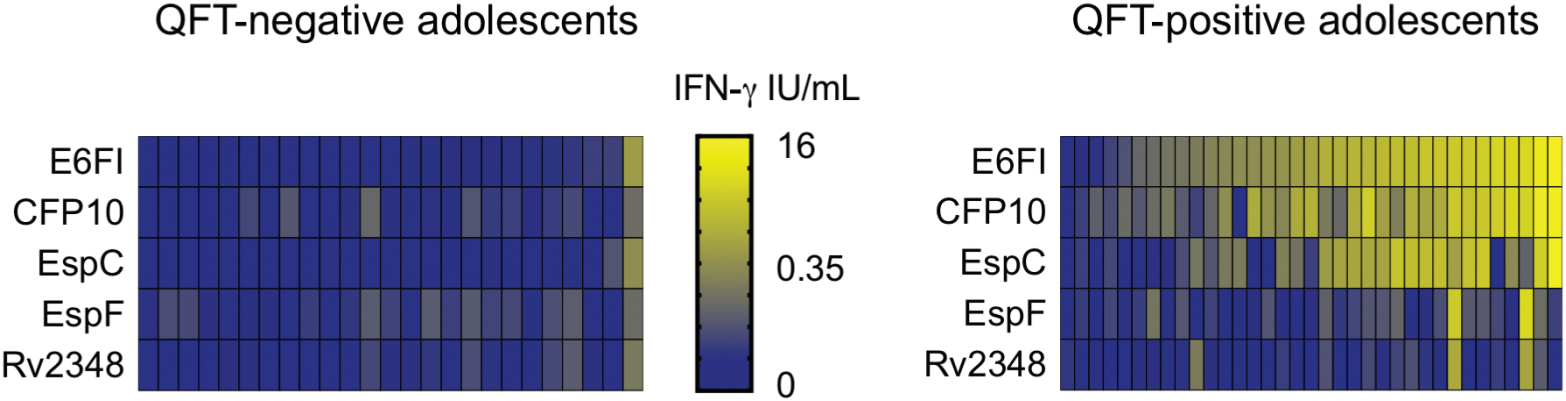
Early secretory antigenic target-6–free IFN-γ release assay antigen recognition by South African adolescents. Heat map of log10(IFN-γ) showing recognition patterns of all antigens in each QFT-negative (left) and QFT-positive (right) participant. In the absence of a cutoff to define recognition of each E6FI antigen, log10(IFN-γ) are presented on a continuous quantitative scale. Abbreviations: IFN-γ, interferon-gamma; QFT, QuantiFERON-TB Gold In-tube.

The concentration of IFN-γ released in response to the ESAT-6–free IGRA antigen cocktail was significantly higher in participants with positive QFT status (QFT+; n = 35) compared to participants with negative QFT status (QFT–; n = 25; Supplementary Figure 2A), and the magnitude of the IFN-γ levels correlated with those induced by QFT (Supplementary Figure 2B). The ESAT-6–free IGRA response was mostly driven by recognition of CFP-10 and EspC (Figure 1).

### Definition of the ESAT-6–Free IGRA Cutoff

**Figure 2.**
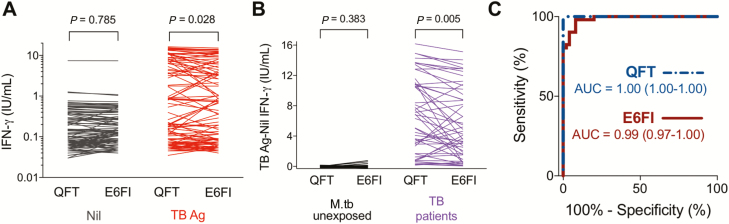
ESAT-6–free IGRA diagnostic accuracy in M.tb-unexposed controls and TB patients. Blood was collected from M.tb-unexposed controls (n = 50, cohort 2a) and microbiologically confirmed TB patients (n = 51, cohort 2b) in QFT and ESAT-6 free IGRA (E6FI) tubes and stimulated for 16–20 hours at 37°C; the IFN-γ concentration was measured by QFT enzyme-linked immunosorbent assay. *A*, Paired analysis (Wilcoxon matched-pairs signed rank test) of IFN-γ values measured upon blood stimulation in QFT and E6FI Nil or TB Ag tubes in all participants. *B*, Paired analysis (Wilcoxon matched-pairs signed rank test) of IFN-γ values measured by QFT and E6FI (TB Ag – Nil) in M.tb-unexposed controls and TB patients. *C*, Receiver operating characteristic analysis of diagnostic accuracy of QFT (dashed line) and E6FI (solid line) assays. Area under the curve and 95% confidence interval are shown. Abbreviations: Ag, antigen; ESAT-6, early secretory antigenic target-6; IFN-γ, interferon-gamma; IGRA, interferon-gamma release assay; M.tb, *Mycobacterium* tuberculosis; QFT, QuantiFERON-TB Gold In-tube; TB, tuberculosis.

The ESAT-6–free IGRA cutoff was determined by comparing M.tb-unexposed participants enrolled in a low TB endemic setting (cohort 2a, n = 50) to microbiologically confirmed TB patients recruited in South Africa (cohort 2b, n = 51; Supplementary Table 1). There was no significant difference in IFN-γ levels following whole blood incubation in QFT or ESAT-6–free IGRA Nil tubes (Figure 2A). Therefore, background IFN-γ values measured in Nil QFT tubes were subtracted from those measured in both QFT TB Ag and ESAT-6–free IGRA TB Ag tubes in all subsequent experiments. In TB patients, the IFN-γ concentrations in the QFT assays (median, 6.79; interquartile range [IQR], 1.70–10.85 IU/mL) were higher than those measured by ESAT-6–free IGRA (median 4.27; IQR, 1.59–9.51 IU/mL; *P* = .005), while equivalent and extremely low IFN-γ concentrations were measured in M.tb-unexposed individuals (Figure 2B). ESAT-6–free IGRA yielded diagnostic accuracy that was comparable to that of QFT for classifying TB-diseased patients vs M.tb-unexposed individuals (Figure 2C). An ESAT-6–free IGRA assay cutoff (0.61 IU/mL) was selected at the IFN-γ concentration that yielded no more than a 5% lower specificity than QFT, as defined in a preset statistical analysis plan. This cutoff resulted in a sensitivity of 82% (Supplementary Figure 3).

### Validation of ESAT-6–Free IGRA Performance in South African Adolescents

**Figure 3.**
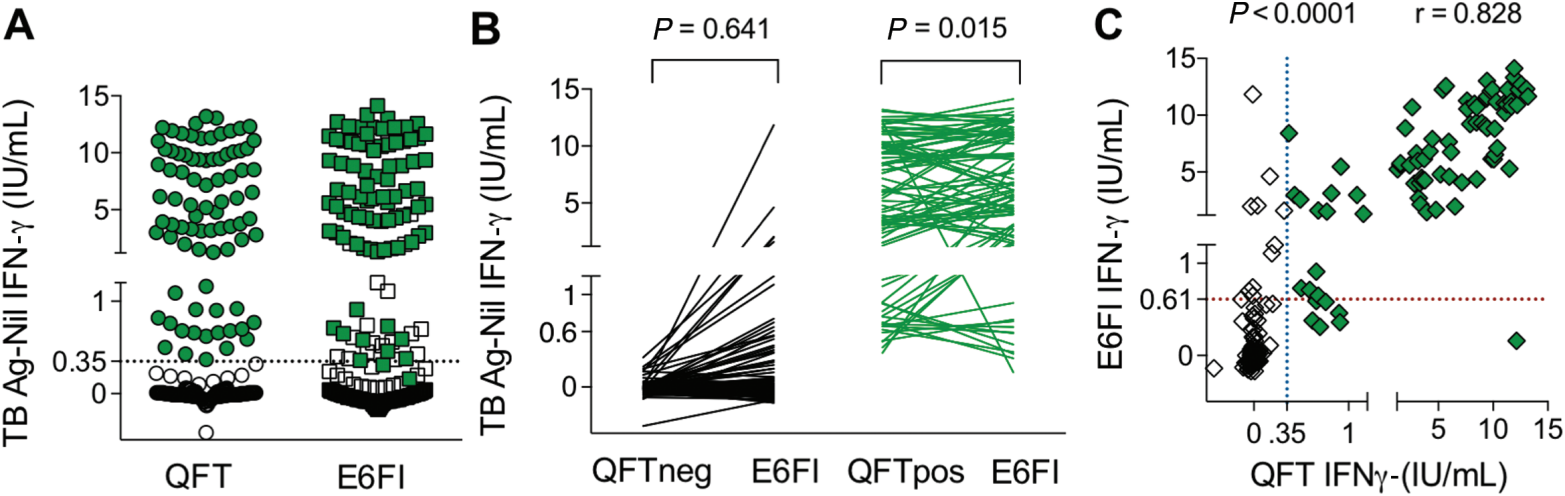
ESAT-6–free IGRA diagnostic accuracy in healthy South African adolescents. Blood was collected from QFT-negative (n = 117, open symbols) and QFT-positive (n = 83, filled symbols) healthy South African adolescents (cohort 3) in QFT and ESAT-6–free IGRA (E6FI) tubes and stimulated for 16–20 hours at 37°C; the IFN-γ concentration was measured by QFT enzyme-linked immunosorbent assay. *A*, Distribution of IFN-γ values measured by QFT and E6FI in all participants. The dotted line denotes QFT cutoff. *B*, Paired analysis (Wilcoxon matched-pairs signed rank test) of IFN-γ values measured by QFT and E6FI (TB Ag – Nil) in QFT-negative and QFT-positive adolescents. *C*, Correlation between IFN-γ values obtained by QFT and E6FI in all participants was assessed using the Spearman test. The dotted lines denote assay cutoffs: 0.35 IU/mL for QFT (vertical line) and 0.61 IU/mL for E6FI (horizontal line). Abbreviations: Ag, antigen; ESAT-6, early secretory antigenic target; IFN-γ, interferon-gamma; IGRA, interferon-gamma release assay; QFT, QuantiFERON-TB Gold In-tube; TB, tuberculosis.

Diagnostic accuracy of ESAT-6–free IGRA for M.tb infection was benchmarked against QFT in an independent cohort of 200 healthy adolescents from South Africa (cohort 3). Median participant age was 14 years (range, 12–18 years), 52% of participants were females, 46% of adolescents self-identified as black African, and 54% were from a mixed ethnic background (Cape mixed ancestry). All participants had received childhood BCG vaccination; none had received a TST within 3 months of recruitment, had previous TB disease nor were known to be exposed to patients with active TB at the time of blood collection; 4% were smokers. Figure 3A shows the distribution of IFN-γ values in adolescents who tested QFT+ (n = 83) or QFT– (n = 117). ESAT-6–free IGRA yielded slightly higher IFN-γ concentrations (median, 6.16; IQR, 2.97–10.91 IU/mL) compared to QFT (median, 5.70; IQR, 1.51–9.93 IU/mL; *P* = .015) in participants who tested QFT+, while no overall difference was observed for those who tested QFT– (Figure 3B). IFN-γ values obtained by ESAT-6–free IGRA and QFT were highly correlated (r = 0.828, *P* < .0001; Figure 3C). Overall, 91% of ESAT-6–free IGRA and QFT results were concordant (kappa = 0.82; 95% CI, 0.74–0.90; McNemar test *P* = .48; Table 1).

**Table 1.** Concordance of ESAT-6–Free IGRA and Quantiferon-TB Gold in Healthy South African Adolescents

		ESAT-6–Free IGRA		
		Negative (n)	Positive (n)	Total (N)
Quantiferon-TB Gold	Negative (n)	106	11	117
	Positive (n)	7	76	83
Total (n)		113	87	200

Abbreviations: ESAT-6, early secretory antigenic target; IGRA, interferon-gamma release assay.

An uncertainty zone to interpret QFT results close to the assay cutoff, falling between 0.2 and 0.7 IU/mL, has been proposed [16–19]. In this adolescent cohort, 5 of 18 (28%) QFT and ESAT-6–free IGRA discordant results fell in the QFT uncertainty zone (Supplementary Figure 4).

### ESAT-6 Free IGRA Variability

To complete the qualification of the ESAT-6–free IGRA assay, we assessed lot-to-lot variation, precision, and stability of ESAT-6–free IGRA blood collection tubes (Supplementary Figure 1B). Experiments were conducted concurrently on blood collected from 12 healthy donors (3 who tested QFT– and 9 who tested QFT+; cohort 1b).

**Figure 4.**
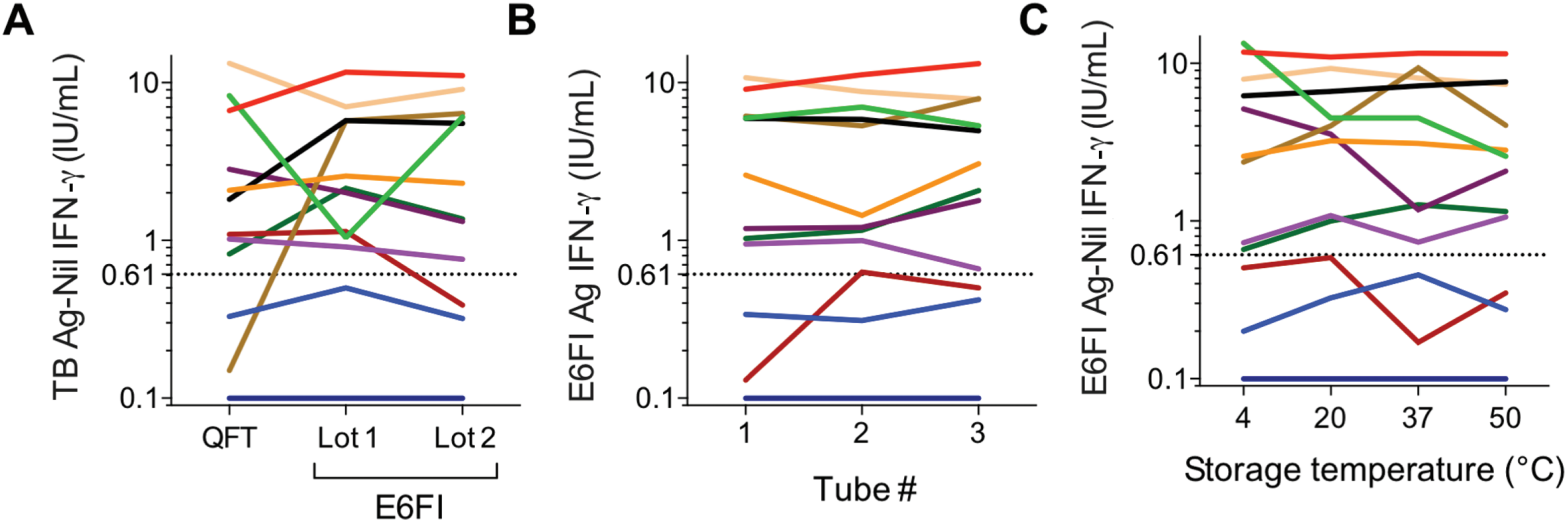
ESAT-6–free IGRA tube variability. Blood was collected from healthy donors (n = 12, cohort 1b) and analyzed as described in Supplementary Figure 1B. IFN-γ values were measured by QFT enzyme-linked immunosorbent assay upon blood stimulation in (*A*) QFT and 2 different lots of ESAT-6–free IGRA (E6FI) tubes (lot-to-lot variability), (*B*) 3 E6FI tubes from lot 2 (precision), and (*C*) 4 E6FI tubes stored at the indicated temperatures for 2 weeks prior to long-term storage at 4°C (stability). Descriptive statistics are shown in Supplementary Figure 5. In all figures, the dotted line denotes the E6FI assay cutoff. Abbreviations: Ag, antigen; ESAT-6, early secretory antigenic target; IFN-γ, interferon-gamma; IGRA, interferon-gamma release assay; QFT, QuantiFERON-TB Gold In-tube; TB, tuberculosis.

No significant difference was observed in IFN-γ values obtained by QFT, ESAT-6–free IGRA lot 1, and ESAT-6–free IGRA lot 2 (*P* > .5 for all comparisons; Figure 4A). Qualitatively, 1 discordant result was observed when QFT was compared to ESAT-6–free IGRA lot 1 and when ESAT-6–free IGRA lot 1 was compared to ESAT-6–free IGRA lot 2, while 2 discordant results were observed when QFT was compared to ESAT-6–free IGRA lot 2 (Figure 4A). Bland-Altman analysis of ESAT-6–free IGRA lot 1 and lot 2 tubes did not show lot-associated systematic bias (bias = –10%; 95% limits of agreement, –123% to 101%), although a high percent difference between lots was observed in some instances (Supplementary Figure 5A), resulting in a concordant correlation coefficient (ρc) = 0.89 (95% CI, 0.68–0.97; Supplementary Figure 5B).

Analysis of blood collected in 3 ESAT-6–free IGRA tubes from lot 2 yielded consistent quantitative and qualitative results, with 1 exception (Figure 4B). Variability observed across replicate ESAT-6–free IGRA assays appeared substantially higher than that due to IFN-γ ELISA intra- and interassay variability (Supplementary Figure 5C).

Storage of ESAT-6–free IGRA blood collection tubes for 2 weeks at 4, 20, 37, or 50°C prior to long-term storage at 4°C did not affect quantitative and qualitative results (Figure 4C) nor did it introduce a systematic bias (Supplementary Figure 5D), although high variability was observed in some samples (Supplementary Figure 5E).

## DISCUSSION

Our aim in this study was to qualify the recently developed ESAT-6–free IGRA, a diagnostic tool for the detection of M.tb infection in individuals vaccinated with ESAT-6–containing TB vaccines. We demonstrate that the 3 antigens substituting ESAT-6 are specific and recognized in healthy individuals with a QFT-positive test. Using a cross-validation design, we defined a cutoff for positive ESAT-6–free IGRA, which is essential to the development of a diagnostic algorithm for test interpretation, and demonstrated that the ESAT-6–free IGRA had diagnostic performance that was comparable to that of QFT in South African adolescents. Also, we explored the variability, precision, and stability of the ESAT-6–free IGRA test and demonstrated that the novel test is sufficiently robust for its intended use in clinical research.

The strength of this analysis was the cross-validation design; this was achieved by determining the cutoff in a population with stringently defined M.tb-unexposed controls and TB cases and validating it in a separate cohort, representative of the target population for the test. In this study, QFT performed as expected both in terms of magnitude of IFN-γ release in samples from TB patients and controls [20] and in terms of positivity rates in the validation cohort. The validation cohort was recruited in a region with extremely high exposure to TB, resulting in about 40% prevalence of QFT+ individuals among adolescents aged 14 years, which is consistent with previous observations in the same community [21].

We observed 9% discordance between ESAT-6–free IGRA and QFT. This finding was expected, as the antigens used in the 2 tests differ and are differentially recognized in different persons [15] (Figure 1). Our findings confirm that EspC is an immunodominant and M.tb-specific antigen and the key support to CFP-10 in the ESAT-6–free IGRA antigen cocktail [13, 15]. Our initial study identified EspF and Rv2348 as important supplement antigens that augment the level of IFN-γ release and extend diagnostic coverage, especially in individuals with latent infection [15]. However, in the population included in this study, these antigens were less frequently recognized. Of note, one third of discordant results fell in the QFT uncertainty zone (IFN-γ values between 0.2 and 0.7 IU/mL) [16]. Our results suggest that the ESAT-6–free IGRA is subject to biological and analytical variability that is similar to that of QFT [16, 22]; therefore, an uncertainty zone should also be established for the ESAT-6–free IGRA.

The development of the ESAT-6–free IGRA arose from the need to detect M.tb infection after vaccination with ESAT-6–containing vaccines. ESAT-6 seems to possess unique properties that drive protective immune responses and has become a central antigen in several vaccine candidates that are in development [23]. However, clinical efficacy trials are required before even the most advanced and promising candidates will be ready for implementation in the field. A robust diagnostic assay to detect M.tb infection after vaccination is critical for the evaluation of the efficacy in preventing M.tb infection in these trials [10], which we showed is feasible in countries with high TB burden and can provide proof of concept to inform further clinical development of vaccine candidates [11].

In TB patients, the magnitude of IFN-γ release was significantly lower in the ESAT-6–free IGRA compared to QFT. While this might have affected the assay cutoff determination, it did not translate into a lower diagnostic sensitivity, determined as positivity rate in the validation cohort.

Regardless, these observations, along with tube-associated variability, suggest a need for improvement of the blood collection tubes. This was addressed through the development of an improved freeze-drying procedure that enhanced peptide solubility. Tubes manufactured through this improved process induced IFN-γ levels equivalent to QFT in individuals who tested QFT+ and lower levels of IFN-γ in controls and will be used in future efficacy studies (Kidola and Ruhwald, manuscript in preparation).

Another limitation in the study was the reliance on active TB cases and uninfected controls to determine the cutoff for a positive test. Although this approach has typically been used to define the IGRA cutoff [20, 24], ideally, endpoints should have been derived from ESAT-6–free IGRA-positive participants who develop active TB in a prospective trial. However, the scale and time required for this endeavor would vastly outweigh the potential benefit to be gained. Further, in the absence of an alternative gold standard to measure M.tb exposure, it is not possible to identify M.tb-unexposed controls in the same high endemic population.

A challenge that limited the generalizability of the blood collection tube variability and stability analysis is that we did not include a direct experimental comparison to QFT. This would allow better referencing to state-of-art assay; however, when compared to the literature [22], the ESAT-6–free IGRA tubes appear robust and field applicable.

In conclusion, we demonstrated that the novel ESAT-6–free IGRA yields a diagnostic accuracy for M.tb infection that is comparable to that of QFT in South African adolescents. The ESAT-6–free IGRA is thus suitable to measure M.tb infection in clinical trials of novel TB vaccine candidates that contain ESAT-6, targeting young, healthy adults.

Further evaluation of the assay needs to be carried out to assess its diagnostic potential among specific subpopulations such as immunocompromised patients and children.

## Supplementary Data

Supplementary materials are available at Clinical Infectious Diseases online. Consisting of data provided by the authors to benefit the reader, the posted materials are not copyedited and are the sole responsibility of the authors, so questions or comments should be addressed to the corresponding author.

ciz034_Suppl_Supplementary_Figure

ciz034_Suppl_Supplementary_Table

## Notes

***Acknowledgments.*** The authors thank all study participants; the South African Tuberculosis Vaccine Initiative, Immunology Research Group, and Statens Serum Institut (SSI) clinical and laboratory teams; Kathryn Rutkowski (AERAS) for statistical input; Marcella Sarzotti-Kelsoe and Daniel Akio Ozaki (QualCon, the Quality Consultants) and Peggy Johnston (the Bill & Melinda Gates Foundation) for input on the assay qualification plan. Elisa Nemes is an International Society for Advancement of Cytometry Marylou Ingram Scholar.

***Financial support.*** This work was supported by AERAS and SSI. The validation cohort was recruited as a substudy of a trial cofunded by AERAS and Sanofi Pasteur. M. R., T. J. S., and G. W. receive funding from European Commission H2020 program (grant TBVAC2020 643381). M. R. receives funding from the Research Council Norway (GLOBVAC 248042/H10). B. S., A. G. L., and G. W. receive funding from the National Institutes of Health (grant 1U01AI115619) and a Strategic Health Innovation Partnership grant from the South African Department of Science and Technology and South African Medical Research Council.

***Potential conflicts of interest.*** E. N., T. J. S., and M. H. report funding from AERAS during the conduct of this study. P. A. has a patent (WO2015090322 A8) pending to the SSI. M. R. and L. L. H. are employed by the SSI, a governmental not-for-profit research organization that holds intellectual property rights on several of the antigens explored in this work for vaccine and diagnostic purposes. All rights are assigned to SSI. A. M. G., R. D. E., and M. D. L. report grants to AERAS from the Bill and Melinda Gates Foundation and the United Kingdom Department for International Development that supported the conduct of the study and shared clinical trial costs with Sanofi Pasteur and GlaxoSmithKline outside the submitted work. All other authors: No reported conflicts. All authors have submitted the ICMJE Form for Disclosure of Potential Conflicts of Interest. Conflicts that the editors consider relevant to the content of the manuscript have been disclosed.

## References

[1] Carpenter C, Sidney J, Kolla R, et al. A side-by-side comparison of T cell reactivity to fifty-nine *Mycobacterium tuberculosis* antigens in diverse populations from five continents. Tuberculosis (Edinb) 2015; 95:713–21. 26277695 10.1016/j.tube.2015.07.001 PMC4666753

[2] Pai M, Denkinger CM, Kik SV, et al. Gamma interferon release assays for detection of *Mycobacterium tuberculosis* infection. Clin Microbiol Rev 2014; 27:3–20. 24396134 10.1128/CMR.00034-13 PMC3910908

[3] Ruhwald M, Aggerbeck H, Gallardo RV, et al; TESEC Working Group Safety and efficacy of the C-Tb skin test to diagnose *Mycobacterium tuberculosis* infection, compared with an interferon γ release assay and the tuberculin skin test: a phase 3, double-blind, randomised, controlled trial. Lancet Respir Med 2017; 5:259–68. 28159608 10.1016/S2213-2600(16)30436-2

[4] Gröschel MI, Sayes F, Simeone R, Majlessi L, Brosch R ESX secretion systems: mycobacterial evolution to counter host immunity. Nat Rev Microbiol 2016; 14:677–91. 27665717 10.1038/nrmicro.2016.131

[5] Brosch R, Gordon SV, Garnier T, et al. Genome plasticity of BCG and impact on vaccine efficacy. Proc Natl Acad Sci U S A 2007; 104:5596–601. 17372194 10.1073/pnas.0700869104 PMC1838518

[6] Aagaard C, Hoang T, Dietrich J, et al. A multistage tuberculosis vaccine that confers efficient protection before and after exposure. Nat Med 2011; 17:189–94. 21258338 10.1038/nm.2285

[7] Lin PL, Dietrich J, Tan E, et al. The multistage vaccine H56 boosts the effects of BCG to protect cynomolgus macaques against active tuberculosis and reactivation of latent *Mycobacterium tuberculosis* infection. J Clin Invest 2012; 122:303–14. 22133873 10.1172/JCI46252 PMC3248283

[8] Hoang T, Aagaard C, Dietrich J, et al. ESAT-6 (EsxA) and TB10.4 (EsxH) based vaccines for pre- and post-exposure tuberculosis vaccination. PLoS One 2013; 8:e80579. 24349004 10.1371/journal.pone.0080579 PMC3861245

[9] Luabeya AK, Kagina BM, Tameris MD, et al; H56-032 Trial Study Group First-in-human trial of the post-exposure tuberculosis vaccine H56:IC31 in *Mycobacterium tuberculosis* infected and non-infected healthy adults. Vaccine 2015; 33:4130–40. 26095509 10.1016/j.vaccine.2015.06.051

[10] Ellis RD, Hatherill M, Tait D, et al. Innovative clinical trial designs to rationalize TB vaccine development. Tuberculosis (Edinb) 2015; 95:352–7. 25802031 10.1016/j.tube.2015.02.036

[11] Nemes E, Geldenhuys H, Rozot V, et al; C-040-404 Study Team Prevention of *M. tuberculosis* infection with H4:IC31 vaccine or BCG revaccination. N Engl J Med 2018; 379:138–49. 29996082 10.1056/NEJMoa1714021 PMC5937161

[12] Arlehamn CS, Sidney J, Henderson R, et al. Dissecting mechanisms of immunodominance to the common tuberculosis antigens ESAT-6, CFP10, Rv2031c (hspX), Rv2654c (TB7.7), and Rv1038c (EsxJ). J Immunol 2012; 188:5020–31. 22504645 10.4049/jimmunol.1103556 PMC3345088

[13] Millington KA, Fortune SM, Low J, et al. Rv3615c is a highly immunodominant RD1 (region of difference 1)-dependent secreted antigen specific for *Mycobacterium tuberculosis* infection. Proc Natl Acad Sci U S A 2011; 108:5730–5. 21427227 10.1073/pnas.1015153108 PMC3078386

[14] Coppola M, van Meijgaarden KE, Franken KL, et al. New genome-wide algorithm identifies novel in-vivo expressed *Mycobacterium tuberculosis* antigens inducing human T-cell responses with classical and unconventional cytokine profiles. Sci Rep 2016; 6:37793. 27892960 10.1038/srep37793 PMC5125271

[15] Ruhwald M, de Thurah L, Kuchaka D, et al. Introducing the ESAT-6 free IGRA, a companion diagnostic for TB vaccines based on ESAT-6. Sci Rep 2017; 7:45969. 28387329 10.1038/srep45969 PMC5384086

[16] Nemes E, Rozot V, Geldenhuys H, et al; C-040-404 Study Team and the Adolescent Cohort Study Team Optimization and interpretation of serial QuantiFERON testing to measure acquisition of *Mycobacterium tuberculosis* infection. Am J Respir Crit Care Med 2017; 196:638–48. 28737960 10.1164/rccm.201704-0817OC PMC5620669

[17] Pai M, Joshi R, Dogra S, et al. Serial testing of health care workers for tuberculosis using interferon-gamma assay. Am J Respir Crit Care Med 2006; 174:349–55. 16690977 10.1164/rccm.200604-472OC PMC2648116

[18] van Zyl-Smit RN, Zwerling A, Dheda K, Pai M Within-subject variability of interferon-g assay results for tuberculosis and boosting effect of tuberculin skin testing: a systematic review. PLoS One 2009; 4:e8517. 20041113 10.1371/journal.pone.0008517 PMC2795193

[19] Zwerling A, Joshi R, Kalantri SP, et al. Trajectories of tuberculosis-specific interferon-gamma release assay responses among medical and nursing students in rural India. J Epidemiol Glob Health 2013; 3:105–17. 23856572 10.1016/j.jegh.2013.03.003 PMC7320393

[20] Ruhwald M, Bodmer T, Maier C, et al; TBNET Evaluating the potential of IP-10 and MCP-2 as biomarkers for the diagnosis of tuberculosis. Eur Respir J 2008; 32:1607–15. 18684849 10.1183/09031936.00055508

[21] Mahomed H, Hawkridge T, Verver S, et al; SATVI Adolescent Study Team Predictive factors for latent tuberculosis infection among adolescents in a high-burden area in South Africa. Int J Tuberc Lung Dis 2011; 15:331–6. 21333099

[22] Tagmouti S, Slater M, Benedetti A, et al. Reproducibility of interferon gamma (IFN-γ) release assays. A systematic review. Ann Am Thorac Soc 2014; 11:1267–76. 25188809 10.1513/AnnalsATS.201405-188OC PMC5469356

[23] Suliman S, Luabeya AKK, Geldenhuys H, et al Dose optimization of H56:IC31 vaccine for TB endemic populations: a double-blind, placebo-controlled, dose-selection trial. Am J Respir Crit Care Med 2019; 199:220–31. 10.1164/rccm.201802-0366OC 30092143

[24] Mori T, Sakatani M, Yamagishi F, et al. Specific detection of tuberculosis infection: an interferon-gamma-based assay using new antigens. Am J Respir Crit Care Med 2004; 170:59–64. 15059788 10.1164/rccm.200402-179OC

