# Supplementary material for: Diagnostic Accuracy of Early Secretory Antigenic Target-6–Free Interferon-gamma Release Assay Compared to QuantiFERON-TB Gold In-tube
Source: Clin Infect Dis. 2019 Jan 21;69(10):1724–30. doi: 10.1093/cid/ciz034 (PMC6821223; doi:10.1093/cid/ciz034)
Supplement: ciz034_Suppl_Supplementary_Figure [file ciz034_suppl_supplementary_figure.pdf]

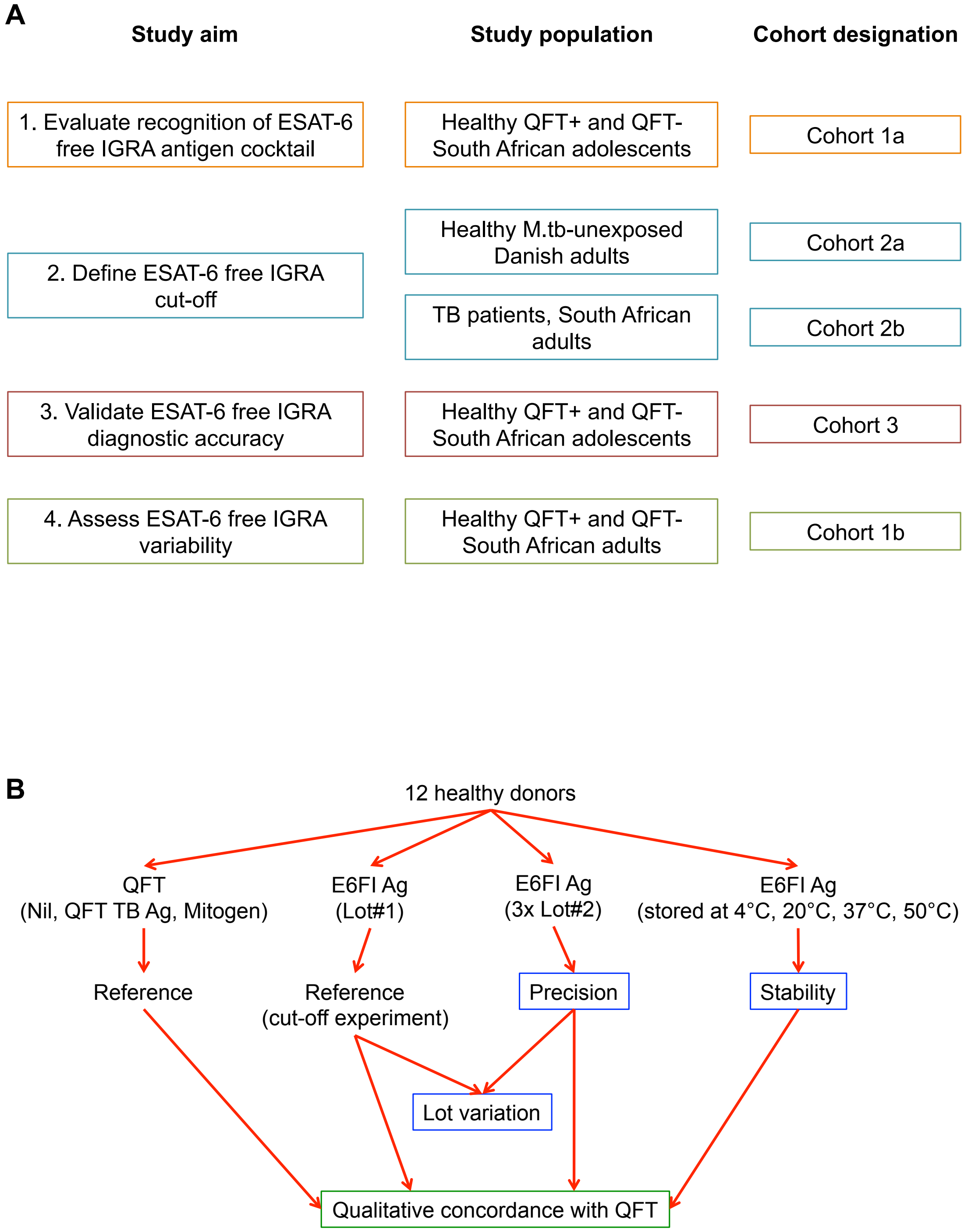

Supplementary Figure 1: Study design.
(A) Study aims were addressed sequentially on independent cohorts of participants.
(B) ESAT-6 free IGRA qualification plan. Blood was collected from healthy donors (n=12, Cohort 1b) directly in Quantiferon-TB Gold (QFT) tubes (Nil, TB Ag, Mitogen) and 8 ESAT-6 free IGRA (E6FI) tubes: 1 tube from lot 1; 3 tubes from lot 2; 4 tubes that had been stored at 4, 20, 37 and 50°C for 2 weeks prior to long term storage at 4°C. Upon incubation at 37°C for 16-20h, IFNγ concentration was measured by QFT ELISA. Precision was assessed by comparing results obtained from 3 identical E6FI tubes (lot 2). Lot variability was assessed between results obtained from lot 1 and the average obtained from lot 2. Stability was assessed across results obtained from tubes stored at different temperatures, considering those stored at 4°C as reference. Concordance of qualitative results obtained from E6FI and QFT was also assessed.

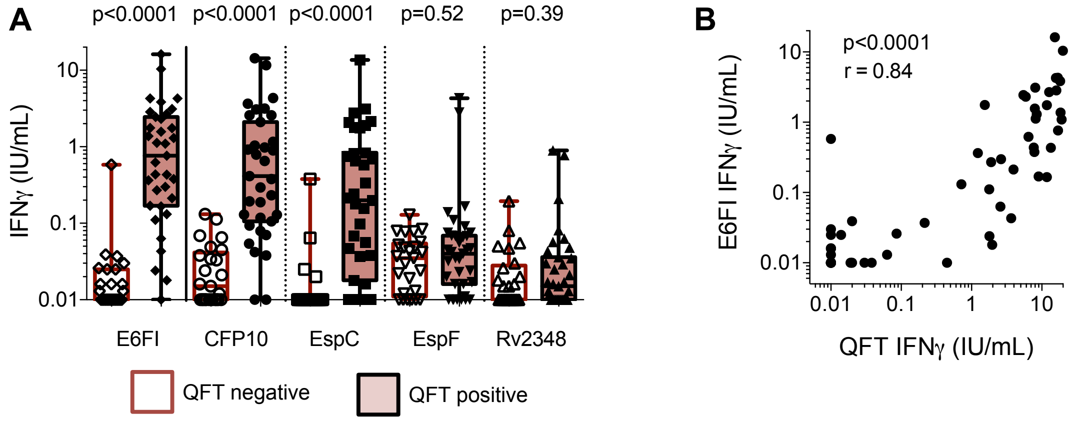

Supplementary Figure 2: ESAT-6 free IGRA antigen recognition by South African adolescents

(A) Whole blood collected from QuantiFERON-TB Gold negative (QFT-; n=25; open symbols) and QFT+ (n=35, filled symbols) adolescents (Cohort 1a) was stimulated with ESAT-6 free IGRA (E6FI) antigen cocktail as well as each of the antigens separately. IFNγ concentration was measured by QFT ELISA. Box and whiskers denote median, inter-quartile range, minimum and maximum values. IFNγ values (Ag - Nil) < 0.01 IU/mL have been set to 0.01 IU/mL to be plotted on a log scale. Results from QFT- and QFT+ adolescents were compared by Mann-Whitney test.

(B) Correlation between IFNγ values obtained by QFT and E6FI in all participants was assessed by Spearman test.

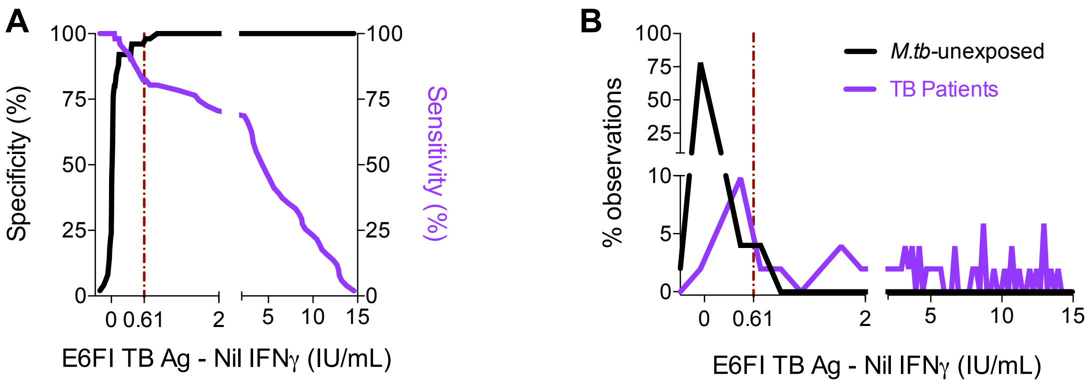

C

	Cases	Controls	Cut-off	Sensitivity (95%CI)	Specificity (95% CI)
QFT+	47	0	0.35	0.92	1
QFT-	4	50	IU/mL	0.81 to 0.98	0.93 to 1.00
E6FI+	42	2	0.61	0.82	0.96
E6FI-	9	48	IU/mL	0.69 to 0.92	0.86 to 1.00

Supplementary Figure 3: ESAT-6 free IGRA cut-off
Blood was collected from M.tb-unexposed controls (n=50, black, Cohort 2a) and microbiologically-confirmed TB patients (n=51, purple, Cohort 2b) in Quantiferon-TB Gold (QFT) and ESAT-6 free IGRA (E6FI) tubes, stimulated for 16-20h at 37°C and IFNγ concentration was measured by QFT ELISA (Figure 2).

(A) E6FI sensitivity and specificity according to different cut-offs, calculated by ROC analysis, shown in Figure 2C.

(B) Distribution of IFNγ values measured by E6FI in M.tb-unexposed controls and TB patients. The vertical dotted line denotes E6FI assay cut-off (0.61 IU/mL).

(C) QFT and E6FI sensitivity and specificity (95% confidence interval) were calculated upon definition of assay cut-offs (standard 0.35 IU/mL for QFT; 0.61 IU/mL for E6FI).

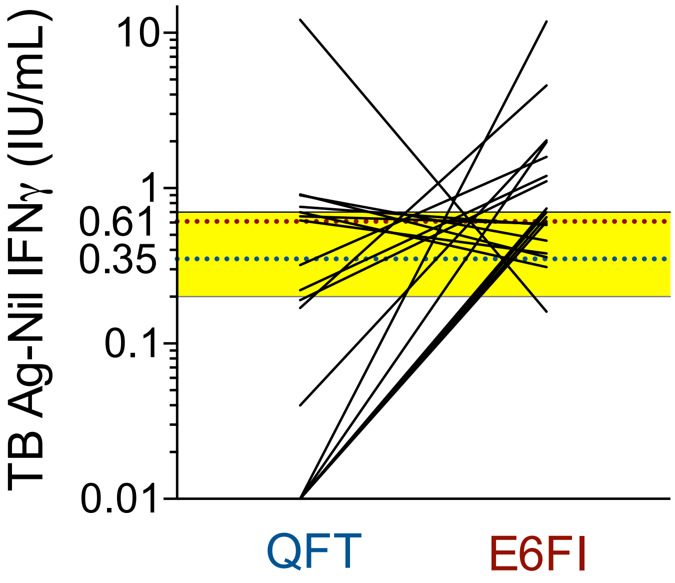

Supplementary Figure 4: QFT uncertainty zone and discordant results

IFNγ values measured by Quantiferon-TB Gold (QFT) and ESAT-6 free IGRA (E6FI) in adolescents (Cohort 3, described in Figure 3 and Table 1) with discordant results. The dotted lines denote assay cut-offs: 0.35 IU/mL for QFT (blue line) and 0.61 IU/mL for E6FI (red line). The yellow area denotes QFT uncertainty zone (0.2-0.7 IU/mL).

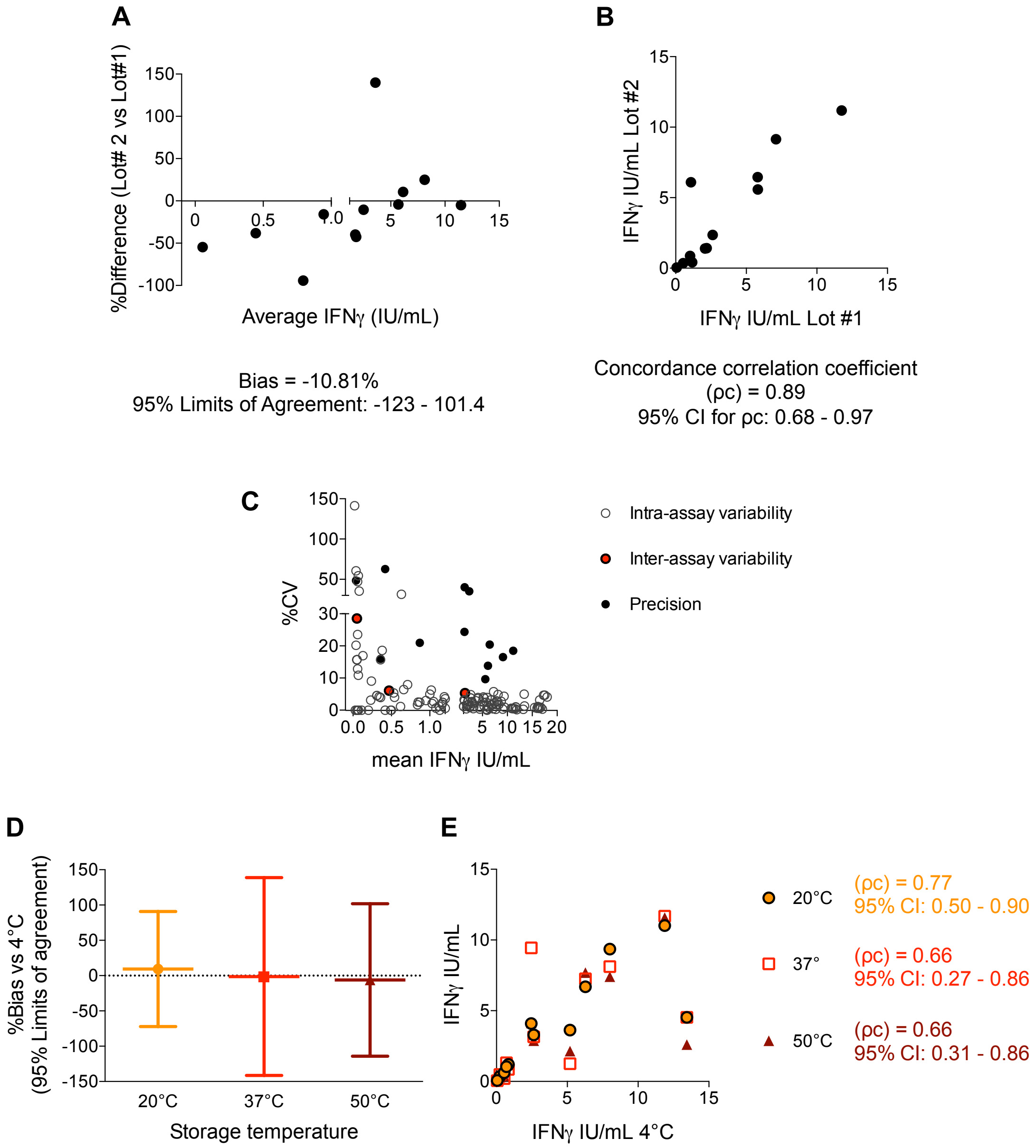

Supplementary Figure 5: ESAT-6 free IGRA tube variability

Blood was collected from healthy donors (n=12, Cohort 1b) and analysed as described in Supplementary Figure 1B.

ESAT-6 free IGRA (E6FI) tubes lot variation was assessed by **(A)** Bland-Altman analysis and **(B)** correlation between results obtained from lot #1 and lot #2 (data from Figure 4A)

(C) Precision of E6FI tubes was assessed by plotting mean IFNγ values measured upon blood incubation in 3 E6FI tubes against their coefficient of variation (%CV) for each participant (data from Figure 4B, black circles). For comparison, mean IFNγ values measured in all individual samples (n=132) used for E6FI qualification experiments (Supplementary Figure 1B and Figure 4) are plotted against the coefficient of variation (%CV) calculated between duplicate ELISA wells, to assess ELISA intra-assay variability (grey circles). Internal quality controls samples (n=3) were included in each ELISA plate (n=4) to assess ELISA inter-assay variability (red circles).

E6FI tube stability was assessed by **(D)** Bland-Altman analysis and **(E)** correlation comparing results from E6FI tubes stored at the indicated temperatures to reference tubes stored at 4°C (data from Figure 4C).
