# Supplementary material for: Diagnostic Accuracy of Early Secretory Antigenic Target-6–Free Interferon-gamma Release Assay Compared to QuantiFERON-TB Gold In-tube
Source: Clin Infect Dis. 2019 Jan 21;69(10):1724–30. doi: 10.1093/cid/ciz034 (PMC6821223; doi:10.1093/cid/ciz034)
Supplement: ciz034_Suppl_Supplementary_Table [file ciz034_suppl_supplementary_table.docx]

**Supplementary Table 1:** **Demographics of M.tb-unexposed controls and TB patients**

|  | | M.tb-unexposed controls  (Cohort 2a, n=50) | TB patients  (Cohort 2b, (n=51) |
| --- | --- | --- | --- |
| Age in years (median, range) | | 37 (26-63) | 41 (18-65) |
| Gender (% males) | | 32 | 69 |
| Ethnicity (%) | Caucasian | 96 | 0 |
|  | Asian | 4 | 0 |
|  | Mixed African | 0 | 98 |
|  | Black African | 0 | 2 |
| BCG vaccination (%) | Yes | 28 | NR |
|  | No | 60 | NR |
|  | Don't know | 12 | NR |
| BCG scar (%) | Yes | NR | 49 |
|  | No | NR | 51 |
| TB diagnosis  (% positive)* | Culture | ND | 76 |
|  | GeneXpert | ND | 88 |
|  | Smear | ND | 2 |
| Previous TB (%) | | 0 | 33 |

* One patient diagnosed by smear microscopy was also confirmed by PCR (line probe assay).

NR = not recorded

ND = not done
